# Osteoporosis Knowledge, Education, and Lifestyle Practices in UAE Women: A Cross-Sectional Study

**DOI:** 10.7759/cureus.104808

**Published:** 2026-03-07

**Authors:** Rand T Jazmati, Alaa J El Taher, Abdolrazzagh S Keramati, Wahg Alenezy, Abdulla Altamimi, Nour Albastaki, Amal Hussein, Asima Karim

**Affiliations:** 1 Medicine and Surgery, University of Sharjah, Sharjah, ARE; 2 Family and Community Medicine, Behavioral Sciences/Public Health, University of Sharjah, Sharjah, ARE; 3 Basic Medical Sciences, University of Sharjah, Sharjah, ARE

**Keywords:** calcium intake, education, lifestyle, osteoporosis, prevention, sun exposure, uae, vitamin d, women, women’s health

## Abstract

Introduction

Osteoporosis is an increasing public health concern in the Gulf region, where vitamin D deficiency and low calcium intake are prevalent. This study aimed to assess osteoporosis knowledge among women in the United Arab Emirates (UAE) and examine its association with educational level and osteoporosis-related lifestyle practices.

Methods

A cross-sectional study was conducted between February and April 2023 among 386 women aged 19-65 years using convenience sampling across community settings in the UAE. Data were collected using a questionnaire assessing demographic characteristics and lifestyle habits, including calcium and vitamin D intake, sun exposure, and physical activity. Osteoporosis knowledge was evaluated using a culturally adapted 17-item Osteoporosis Knowledge Assessment Tool (OKAT). Descriptive and bivariate analyses were performed.

Results

The mean OKAT score was 7.39 ± 3.05, with most participants demonstrating average ‎knowledge. Age was significantly associated with knowledge levels (χ²(4) = 20.201, p < 0.001, ‎Cramér’s V = 0.162), with older participants more likely to have higher knowledge, while no ‎statistically significant association was observed between education level and knowledge (χ²(4) ‎‎= 3.854, p = 0.426, Cramér’s V = 0.071) or ethnicity and knowledge (χ²(4) = 5.776, p = 0.217, ‎Cramér’s V = 0.086). Knowledge was significantly associated with both vitamin D ‎supplementation (p = 0.002, Cramér’s V = 0.148) and calcium supplement intake (p = 0.006, ‎Cramér’s V = 0.137). Preventive lifestyle behaviors were generally suboptimal‎.

Conclusion

In this sample, osteoporosis knowledge was significantly associated with vitamin D and calcium supplementation but not with broader preventive behaviors such as dietary calcium intake or sun exposure. Due to the cross-sectional design and convenience sampling, findings should be interpreted as associative and not causal. Further research is needed to better understand factors influencing osteoporosis prevention behaviors.

## Introduction

Osteoporosis is a systemic skeletal disorder characterized by microstructural deterioration, ‎impaired bone strength, and an increased propensity to fractures. The World Health ‎Organization ‎(WHO) clinically defines osteoporosis as a bone mineral density (BMD) T-score ≤ -2.5 SD below ‎the mean for healthy, young adult women [1]‎. Osteoporosis is a silent condition, meaning bone ‎weakening progresses unnoticed until a fracture occurs unexpectedly [2], making it a major ‎global public health concern [3]. While affecting individuals of any age, osteoporosis ‎disproportionately impacts females; an estimated 200 million women worldwide are affected ‎‎[4]. Approximately 40-50% of women experience osteoporotic fractures in their lifetime [1,5].‎ The burden of osteoporosis extends beyond fractures, significantly impacting Health-‎Related Quality of Life (HRQoL), due to functional impairment, prolonged immobilization, and ‎restrictions in activities of daily living [6,7]. Therefore, it is essential to improve HRQoL ‎through effective preventive practices for osteoporosis. Research indicates a significant correlation between osteoporosis knowledge and beliefs and adoption of osteo-protective ‎practices [3], suggesting that education may be associated with behavior via increased knowledge. This highlights the critical importance of primary healthcare professionals in ‎delivering effective health education programs that empower women to recognize the vague and non-specific symptoms of osteoporosis and adopt preventive measures [8].

Knowledge gaps are especially evident in Gulf countries, where recent reports show ‎poor adherence to preventive behaviors despite rising awareness levels [9]. A ‎2024 survey conducted in the United Arab Emirates (UAE) showed only 41.9% of adults ‎demonstrated good osteoporosis knowledge, while 45.3% showed poor preventive practices [10], indicating that awareness alone does not guarantee behavior change.

However, existing literature presents multiple limitations regarding the assessment of ‎osteoporosis knowledge. Many studies lack a comprehensive exploration of factors, such as ‎socioeconomic status, access to exercise facilities, and dietary habits. Furthermore, ‎methodological weaknesses, including small sample sizes, often undermine the validity of their ‎findings. While osteoporosis is a global health issue, the UAE possesses unique lifestyle, ‎cultural, and environmental factors that influence both awareness and preventative behaviors ‎‎[11]. A 2020 regional report estimated osteoporosis prevalence at 3.1% in the UAE, with ‎postmenopausal Saudi women showing rates up to 39.5%, reflecting a significant GCC burden ‎‎[12].

To date, research specifically assessing osteoporosis knowledge among the UAE population is scarce, and the pathway linking educational attainment to knowledge and, in turn, to preventive behaviors remains poorly understood, particularly among younger women. The primary objective of this study is to assess osteoporosis knowledge levels among women in the UAE, while the secondary objectives are to examine the association between educational attainment and knowledge, and between knowledge and preventive lifestyle practices. We hypothesize that higher educational attainment is associated with greater osteoporosis knowledge, which in turn is linked to more consistent adoption of preventive behaviors. Given the cross-sectional study design, analyses are intended to identify associations rather than infer causality. We conceptualize educational attainment as influencing osteoporosis knowledge, which in turn affects preventive lifestyle practices, framing knowledge as a potential mediator between education and behavior. Therefore, the present study aims to provide region-specific evidence to inform tailored public health interventions that effectively bridge the knowledge-behavior gap.

## Materials and methods

‎Study design and sampling‎

A cross-sectional study was conducted over a two-month period, from February 2023 to April ‎‎2023. Our study included females aged 18 to 65 years old residing in the UAE, who are able to understand Arabic or English. We excluded participants outside the specified age range, women currently studying or working in healthcare, and those with a prior diagnosis of ‎osteoporosis. Incomplete questionnaires were also excluded. A minimum sample ‎size of 365 was calculated based on a 5% margin of error and a 50% expected prevalence. Non-‎probability convenience sampling was employed due to ease of access to the target ‎population‎.

‎Study tool

The original OKAT consists of 20 items designed to assess knowledge regarding osteoporosis ‎risk factors, prevention, and treatment [13]. Our study used a modified version of the ‎OKAT, with minor modifications made by the ‎authors for cultural relevance [13]. For the present study, three items were removed, including race-‎related and Australia-specific questions that were not applicable to the UAE population. In addition, selected statements were reworded for clarity, and one culturally adapted item ‎addressing treatment availability in the UAE was added. Certain less relevant risk factors (e.g., ‎alcohol consumption) were excluded to enhance contextual relevance. The final modified ‎instrument consisted of 17 true or false questions designed to evaluate a participant’s ‎knowledge of osteoporosis, including risk factors, consequences, and preventative measures. The ‎theoretical maximum score for the modified OKAT is 17. In the present sample, the observed ‎score range was 0-14, reflecting the knowledge levels of the study population rather than the ‎ceiling of the instrument itself. Participants were categorized into low (0-5), average (6-11), ‎and high (12-17) knowledge level groups based on the theoretical score range. The internal ‎consistency of the modified OKAT in this sample was good (Cronbach’s α = 0.849). The OKAT ‎has been validated and reliably used in previous studies to assess a population’s knowledge of ‎osteoporosis [13]. The questionnaire was available in both Arabic and English, requiring participants to be efficient in either language. The questionnaire was pilot-tested with 20 participants ‎from the medical field, including medical students and faculty from the College of Medicine, to ‎assess the clarity, comprehensibility, and the time required for completion. The final ‎questionnaire consisted of 32 closed-ended questions divided into three sections: demographics(age, education, ethnicity, etc.), osteoporosis knowledge (risk factors, symptoms, prevention, and ‎treatment), and osteoporosis-preventative practices (e.g., Ca intake, exercise, etc.). The final modified questionnaire is provided in Appendix A (English version) and Appendix B (Arabic version).

‎Data collection ‎

**Figure 1:**
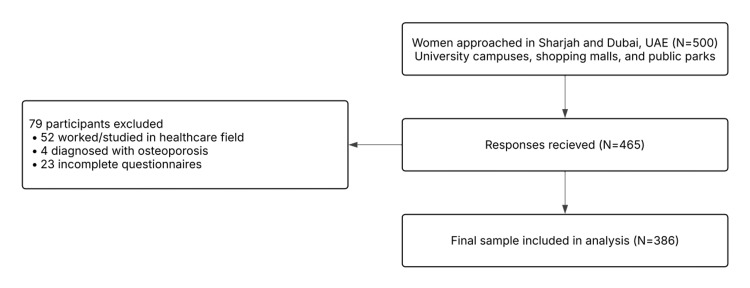
Recruitment flow diagram illustrating participant selection and exclusion criteria

Data collection commenced immediately after obtaining ethical approval and finalizing the ‎sample size calculation and study tools. Self-‎administered questionnaires were distributed in the form of a QR code to eligible female ‎participants encountered during the data collection period. The questionnaire was digitized ‎using Google Forms (Google LLC, Mountain View, CA, USA), allowing participants to complete it electronically via their personal ‎devices. Both the Arabic and English versions of the questionnaire were provided. Recruitment ‎took place in university campuses, public parks, and shopping malls located in Sharjah and ‎Dubai, UAE. The exclusion criteria were tested whilst the participants filled out the questionnaire; those who fit the exclusion criteria were automatically redirected, preventing them from completing the survey. Only participants who met the inclusion criteria ‎and completed the questionnaire were included in the study. The study was approved by the ‎University of Sharjah Research Ethics Committee (approval no. REC-23-02-20-01-S). Participation was completely voluntary and ‎done with informed consent through a participant information sheet. Participants remain ‎anonymous, and their responses are only used for the study’s purpose. Data collected through ‎Google Forms was password-protected and only accessible by the study’s conductors. The raw ‎questionnaire data were then transferred onto Microsoft Excel (Microsoft Corp., WA, USA) for initial processing, and IBM SPSS Statistics (IBM Corp., Armonk, NY) was used for further analysis of the data. A total of 500 eligible individuals were ‎invited to participate in the study. Of these, 386 completed the questionnaire, yielding a ‎response rate of 77.2%. All completed questionnaires were included in the final analysis. A ‎detailed recruitment flow diagram is presented in Figure 1. 

‎Data analysis

Data analysis was performed using IBM SPSS Statistics version 28. Univariate analysis was conducted ‎using descriptive statistics, including measures to condense data (frequency and relative ‎frequency), measures of central tendency, and measures of variability (standard ‎deviation). Bivariate analysis was performed to compare two variables depending on the type ‎of data. Chi-square tests (for categorical variables), t-tests, and Pearson correlation analyses ‎were performed using a significance level of 0.05. ‎Effect sizes were calculated using Cramér’s V for chi-square tests. A multinomial logistic ‎regression was conducted to examine the independent associations between demographic ‎variables (age, education level, and ethnicity) and knowledge score category (low, average, ‎high), with average knowledge as the reference outcome and adults aged 19-35 as the reference ‎age group. Results are reported as odds ratios (OR) with 95% confidence intervals (CI).‎ For analysis, education responses were recoded into three categories: secondary (did not complete high school and high school diploma combined), undergraduate (bachelor's), and postgraduate (master's and PhD combined). Ethnicity responses were recoded into three categories: UAE national, Arabs (except UAE), and non-Arabs (Far East Asian, Southeast Asian, Caucasian, and other combined).

## Results

‎Study population demographics ‎

The study involved 386 participants with a mean age of 37.2 years. Table 1 presents ‎demographics of the participants, including age, education level, and nationality. 

**Table 1:** Study population demographics and their association with osteoporosis knowledge level‎ Percentages within each demographic group represent row percentages. Associations were assessed using chi-square ‎test. Age: χ² = 20.201, df = 4, p < 0.001, Cramér’s V = 0.162. Educational level: χ² = 3.854, df = 4, p = 0.426, ‎Cramér’s V = 0.071. Ethnicity: χ² = 5.776, df = 4, p = 0.217, Cramér’s V = 0.086‎.

Dependent variables	Total (N = 386), n (%)		Low knowledge, n (%)	Average knowledge, n (%)	High knowledge, n (%)	P-value
Age	19-35	191 (49.5%)	59 (30.9%)	125 (65.4%)	7 (3.7%)	<0.001
	36-50	140 (36.3%)	39 (27.9%)	84 (60%)	17 (12.1%)	
	51-65	55 (14.2%)	6 (10.9%)	39 (70.9%)	10 (18.2%)	
Educational level	Secondary education	92 (23.8%)	29 (31.5%)	55 (59.8%)	8 (8.7%)	0.426
	Undergraduate	213 (55.2%)	59 (27.7%)	134 (62.9%)	20 (9.4%)	
	Postgraduate	81 (21%)	16 (19.8%)	59 (72.8%)	6 (7.4%)	
Ethnicity	UAE national	127 (32.9%)	31 (24.4%)	89 (70.1%)	7 (5.5%)	0.217
	Arabs (except UAE)	198 (51.3%)	53 (26.8%)	122 (61.6%)	23 (11.6%)	
	Non-arabs	61 (15.8%)	20 (32.8%)	37 (60.7%)	4 (6.6%)	

Age was significantly associated with knowledge level, with a small-to-moderate effect size (Cramér’s V = 0.162, p < 0.001). In contrast, educational level (Cramér’s V = 0.071, p = 0.426) and ethnicity (Cramér’s V = 0.086, p = 0.217) were not significantly associated with knowledge category (Table 1). High knowledge increased progressively across age groups (3.7% among 19-35 years, 12.1% among 36-50 years, and 18.2% among 51-65 years), while low knowledge was most prevalent in the youngest group (30.9%) and least prevalent in the oldest (10.9%). Most participants were undergraduates (55.2%), followed by those with secondary (23.8%) and postgraduate education (21.0%). The largest ethnic group was non-UAE Arabs (51.3%), followed by UAE nationals (32.9%) and non-Arabs (15.8%). Full distributions are presented in Table 1.

Multinomial logistic regression

A multinomial logistic regression was conducted to examine the predictors of osteoporosis ‎knowledge category (low, average, high), with average knowledge as the reference outcome ‎category and adults aged 19-35 as the reference age group. Age, education level, and ethnicity ‎were entered as predictors.‎

Age was the only significant predictor of the knowledge category. Adults aged 51-65 had significantly higher odds of being classified in the high knowledge category compared to those ‎aged 19-35 (OR = 5.084, 95% CI (1.769, 14.605), p = 0.003). Adults aged 36-50 also had ‎significantly higher odds of high knowledge compared to those aged 19-35 (OR = 3.257, 95% ‎CI (1.266, 8.380), p = 0.014). Education level was not a significant predictor of high knowledge ‎across any category (all p > 0.05). Similarly, ethnicity did not significantly predict high ‎knowledge category membership (all p > 0.05). These findings reinforce the chi-square results ‎and confirm that age is the primary demographic determinant of osteoporosis knowledge in this ‎sample. Full regression results are presented in Table 2‎.

**Table 2:** Multinomial logistic regression - predictors of high osteoporosis knowledge (reference outcome: average knowledge) ‎ᵃ Reference category. OR = odds ratio; CI = confidence interval. Reference outcome: average knowledge (2.00). Age reference ‎group: 19–35 (newage = 3.00). Education reference group: Postgraduate. Ethnicity reference group: UAE national‎

Knowledge category	Predictor (vs. reference)	OR (95% CI)	p-value
High knowledge	Age: 51–65 vs. 19–35ᵃ	5.084 (1.769–14.605)	0.003
	Age: 36–50 vs. 19–35ᵃ	3.257 (1.266–8.380)	0.014
	Education: Secondary vs. Postgraduateᵃ	1.832 (0.568–5.909)	0.311
	Education: Undergraduate vs. Postgraduateᵃ	1.661 (0.584–4.720)	0.341
	Ethnicity: Arabs (except UAE) vs. UAE nationalᵃ	2.299 (0.920–5.746)	0.075
	Ethnicity: Non-Arabs vs. UAE nationalᵃ	1.445 (0.381–5.479)	0.589
Low knowledge	Age: 51–65 vs. 19–35ᵃ	0.348 (0.138–0.876)	0.025
	Age: 36–50 vs. 19–35ᵃ	0.982 (0.590–1.634)	0.944
	Education: Secondary vs. Postgraduateᵃ	1.880 (0.907–3.897)	0.089
	Education: Undergraduate vs. Postgraduateᵃ	1.536 (0.796–2.963)	0.200
	Ethnicity: Arabs (except UAE) vs. UAE nationalᵃ	1.240 (0.729–2.108)	0.427
	Ethnicity: Non-Arabs vs. UAE nationalᵃ	1.723 (0.854–3.475)	0.128

Knowledge level regarding osteoporosis ‎

**Figure 2:**
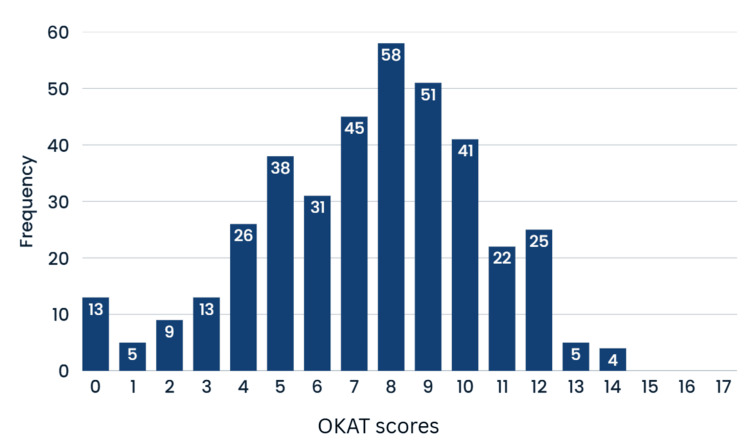
Histogram of the Osteoporosis Knowledge Assessment Tool (OKAT) scores of the study participants ‎ Mean = 7.39‎; standard deviation = 3.054‎; N = 386‎

Figure 2 represents the distribution of osteoporosis knowledge scores among ‎participants. 

The ‎mean score was 7.39 ± 3.054. Most of the participants, 64.2% (n = 248), had an average score, ‎while 26.9% (n=104) had a low OKAT score and 8.8% (n = 34) had a high score.

‎Osteoporosis-related living habits ‎

**Figure 3:**
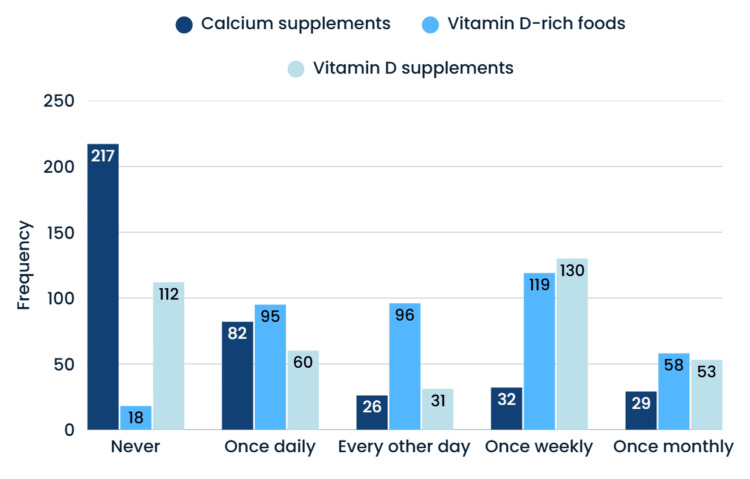
Bar chart of the frequency of calcium and vitamin D intake among the participants ‎Vitamin D-rich foods: liver, mushrooms, spinach, fish oils, eggs

Analysis of calcium and vitamin D supplementation patterns is shown in Figure 3. 

Calcium supplements showed the highest rate of non-use (217 participants never ‎took calcium supplements). Daily intake was 82 participants for calcium supplements, 95 for vitamin ‎D-rich foods, and 60 for vitamin D supplements. Weekly consumption was most common for vitamin D, ‎with 119 participants consuming vitamin D-rich foods and 130 taking supplements. This pattern ‎suggests relatively higher adherence to vitamin D intake recommendations compared to calcium ‎supplementation.‎

‎Knowledge and calcium supplementation ‎

**Figure 4:**
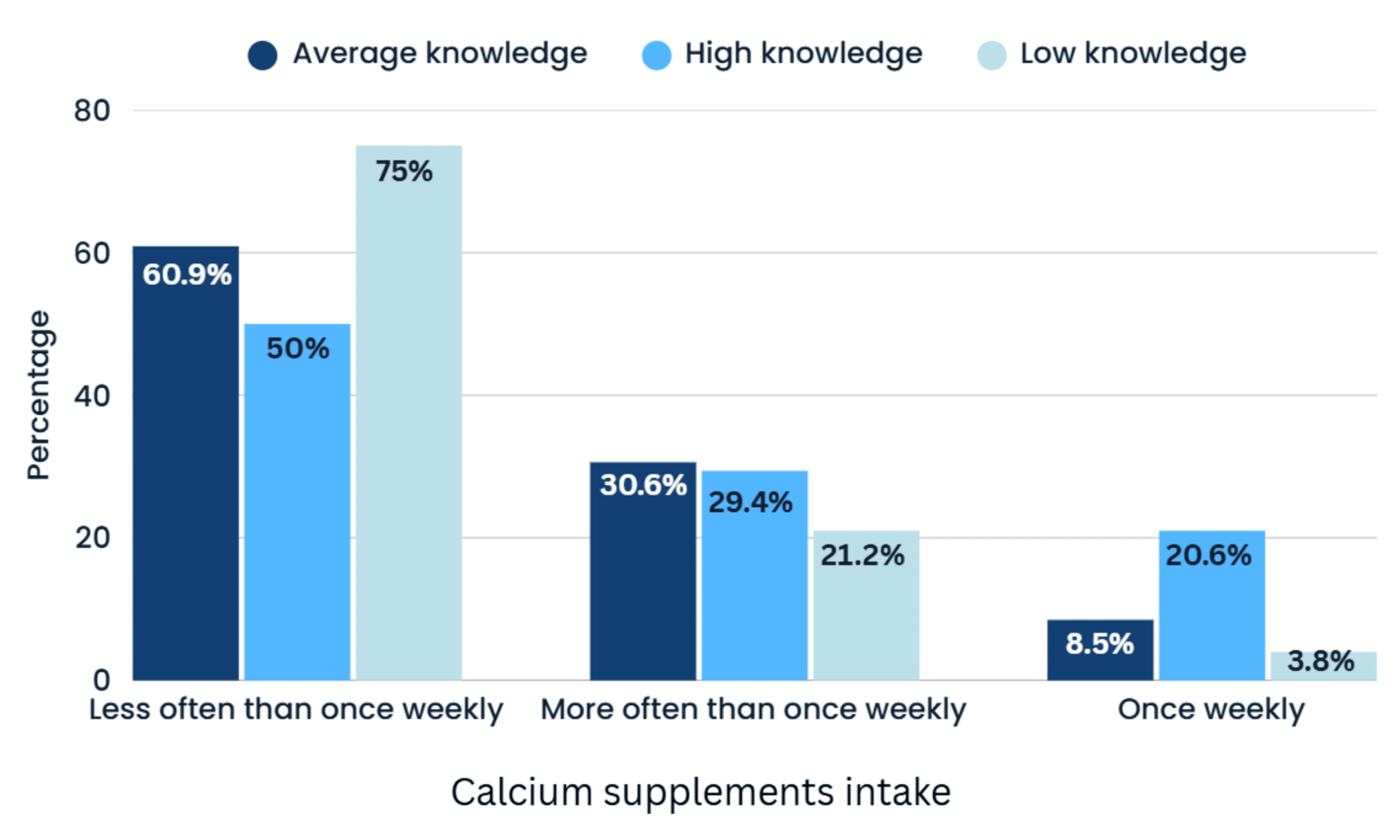
Bar chart of the correlation between knowledge level and intake of calcium supplements‎ Chi-square test values: χ² = 14.469, df = 4, p = 0.006, V = 0.137

Figure 4 illustrates the relationship between osteoporosis knowledge levels and calcium ‎intake (Chi-square test). 

Among individuals with high knowledge, 50.0% took calcium supplements ‎less than once a week, 20.6% once a week, and 29.4% more often than once a week. Among ‎individuals with low knowledge, 75.0% took calcium supplements less than once a week, 3.8% ‎once a week, and 21.2% more often. The chi-square test yielded a statistically significant result ‎‎(p = 0.006), indicating that higher osteoporosis knowledge was associated with more frequent ‎calcium supplement intake.

‎Knowledge and vitamin D supplementation

**Figure 5:**
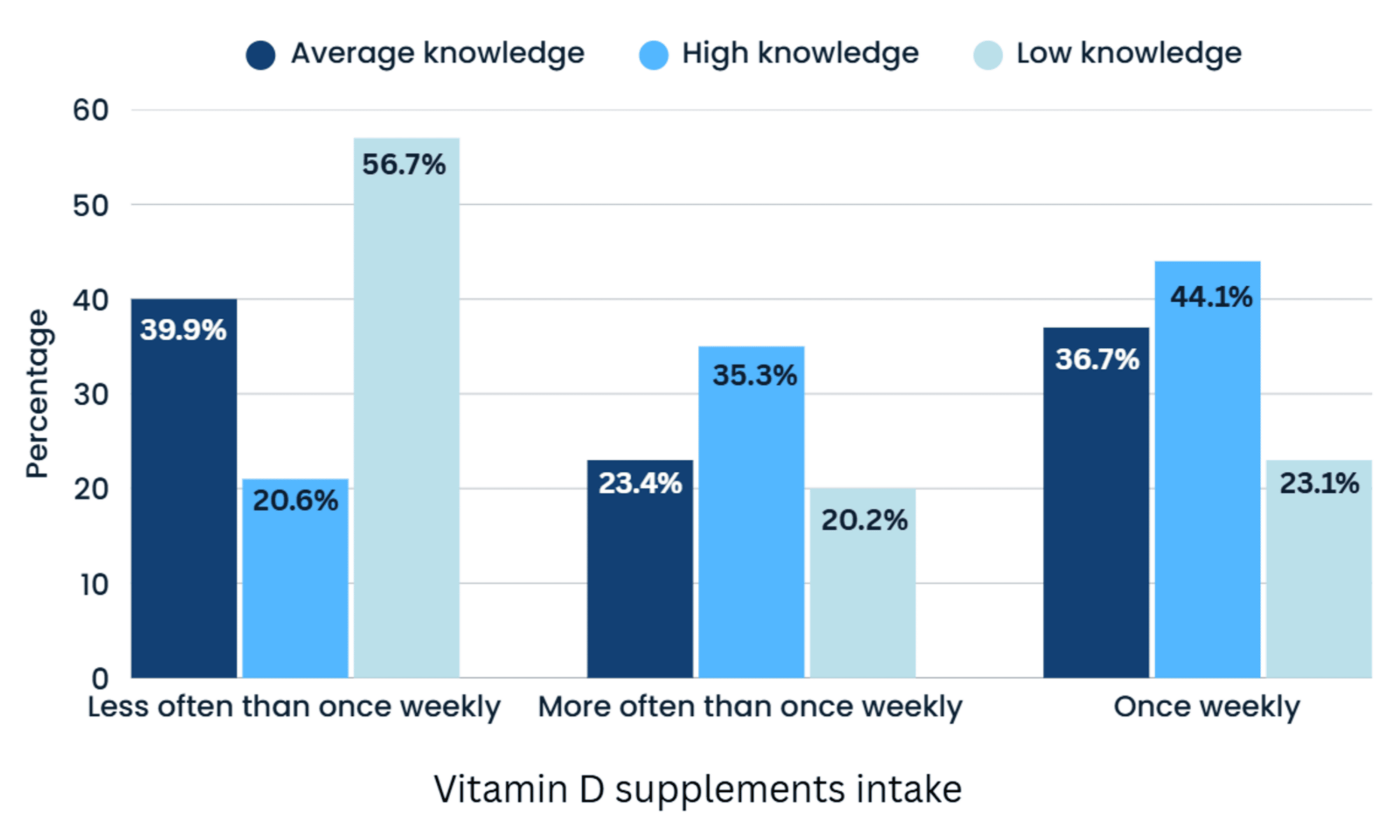
Bar chart of the correlation between knowledge level and intake of vitamin D supplements Chi-square test values: χ² = 16.856, df = 4, p = 0.002, ‎Cramér’s V = 0.148

Figure 5 illustrates the association between osteoporosis knowledge levels and vitamin D ‎supplement intake (Chi-square test). 

Among individuals with high knowledge, 44.1% took vitamin D supplements ‎once a week, 20.6% less often than once a week, and 35.3% more than once a week. Among ‎individuals with low knowledge, 56.7% took vitamin D supplements less often than once a week, ‎‎20.2% once a week, and 23.1% more than once a week. The chi-square test yielded a ‎statistically significant result (p = 0.002), indicating that higher osteoporosis knowledge was ‎associated with more regular Vit D supplement intake.

‎Milk consumption and barriers to calcium intake

The study revealed concerning patterns in milk consumption, with most participants ‎‎(52.59%) reporting no daily milk intake. Among milk consumers, 42.75% consumed one to two glasses ‎per day, while only 4.66% consumed more than two glasses daily. This low level of milk ‎consumption is concerning, given milk's role as a primary dietary source of calcium.‎ The participants reported several barriers to adequate calcium intake. The most frequently ‎reported issue was unclear dietary requirements (35.75%), followed by lactose intolerance ‎‎(13.99%), supplement unavailability (8.03%), medication interactions (5.96%), and other ‎unspecified reasons (36.27%). These findings highlight the need for clearer ‎public guidance regarding calcium sources and recommended intake levels.

‎Sun exposure and vitamin D-rich dietary intake

Sun exposure analysis revealed that most participants (59.33%) reported getting up to ‎‎15 minutes of daily sun exposure, which is below recommended levels for adequate natural Vit D synthesis, suggesting that sun exposure remains a suboptimal source of Vit D in this population.

‎Exercise motivation and interest in preventive education

Analysis of exercise motivation revealed that gym location was the primary ‎motivational factor (45.6%), followed by social media‎ influence (33.68%). In contrast, public ‎health campaigns had the least impact, motivating only 20.73% of participants. Despite the limited impact of awareness campaigns, there was a strikingly strong interest in ‎preventive education. A large majority of participants (88.08%) expressed a desire for more ‎osteoporosis prevention programs in the UAE, highlighting a key opportunity to enhance ‎outreach through more accessible and engaging platforms‎.

## Discussion

Osteoporosis is a growing public health concern in the Gulf region, where vitamin D deficiency ‎and low calcium intake are highly prevalent. In this study, women in the UAE demonstrated ‎average levels of osteoporosis knowledge. Knowledge was significantly associated with both ‎vitamin D supplementation and calcium supplement intake, suggesting that higher knowledge ‎levels are linked to greater engagement with supplementation behaviors. However, absolute ‎supplementation rates remained low across all knowledge groups, and these associations did not ‎extend uniformly to all preventive behaviors; over half of the participants reported never using ‎calcium supplements, only 42.8% consumed milk daily, and most had limited sun exposure. These findings suggest that while knowledge may be associated with supplementation practices, other barriers impede broader preventive behavior adoption.‎

Vitamin D supplementation showed a statistically significant association with higher knowledge ‎levels (p = 0.002, Cramér’s V = 0.148), as did calcium supplement intake (p = 0.006, Cramér’s ‎V = 0.137). Nearly 90% of participants also expressed interest in osteoporosis prevention ‎initiatives, suggesting potential for improved preventive behaviors with targeted support. ‎Similar patterns have been observed in previous UAE studies, where only 41.9% of adults ‎demonstrated good osteoporosis knowledge, and 45.3% reported poor preventive practices [10]‎. ‎

Comparable trends have been reported across the region. In Riyadh, although 90.5% of young adults believed that calcium-rich foods could prevent osteoporosis, only 35.7% consumed them adequately [14]. Likewise, studies in Jazan and among Saudi females attending primary care clinics showed that osteoporosis knowledge was not consistently associated with preventive behaviors [15]. Similar findings were also reported among UAE university students, where moderate awareness did not correspond with adequate calcium intake [16]. These results suggest that knowledge alone may not be sufficient to support calcium-related behavior change. Notably, 59.3% of participants in the present study reported sun exposure of 15 minutes or less per day, reflecting lifestyle barriers previously reported in Saudi Arabia and Syria, despite awareness of vitamin D benefits [17-20].‎‎ Age was significantly associated with higher knowledge levels (p < 0.001), with older ‎participants demonstrating a higher likelihood of high osteoporosis knowledge. This was ‎confirmed in the multinomial logistic regression, where adults aged 51-65 had approximately ‎five times higher odds of being in the high knowledge category compared to those aged 19-35 ‎‎(OR = 5.084, 95% CI (1.769, 14.605), p = 0.003), and adults aged 36-50 had approximately three ‎times higher odds (OR = 3.257, 95% CI (1.266, 8.380), p = 0.014). These findings suggest that ‎accumulated life experience and increased health exposure with age may be associated with ‎greater osteoporosis knowledge.‎

By contrast, no statistically significant association was observed between educational level and ‎knowledge category (p = 0.426, Cramér’s V = 0.071), and no significant association was found ‎between ethnicity and knowledge (p = 0.217, Cramér’s V = 0.086). These findings suggest that ‎demographic factors beyond age do not independently explain knowledge differences in this ‎sample‎. ‎ Exercise behavior in this study appeared largely convenience-driven, with 45.6% of ‎‎participants citing gym location as the main motivator and only 20.7% influenced by public ‎‎health campaigns. Poor preventive practices have similarly been reported among Saudi females, where only 20% reported always consuming calcium-rich foods, and 14% consistently obtained adequate sun exposure [21]. ‎

Encouragingly, 88.1% of participants expressed interest in osteoporosis prevention programs. ‎This finding is consistent with reports from Bahrain and Jordan, where awareness has improved ‎modestly, but sustained behavior change remains inconsistent [22-23]. Overall, these findings ‎suggest that higher osteoporosis knowledge was associated with both vitamin D and calcium ‎supplementation, while other preventive behaviors, such as dietary calcium intake and sun ‎exposure, remained suboptimal regardless of knowledge level. The high expressed interest in ‎prevention programs may represent an opportunity to explore targeted behavior change ‎approaches in future interventions, though the effectiveness of such strategies would need to be ‎evaluated in prospective studies‎.‎

Strengths and limitations

A key strength of this study is the consistent and reliable measurement of knowledge and behavioral practices. The internal consistency of the modified OKAT was good (Cronbach's α = 0.849), supporting the reliability of the knowledge measure. Additionally, the inclusion of both knowledge and behavioral measures provided a more comprehensive view of the knowledge-behavior relationship in osteoporosis prevention.

However, several limitations should be noted. First, reliance on self-reported data may have ‎introduced bias, especially regarding supplement use, exercise, and sun exposure. Second, the ‎use of convenience sampling via QR code in public spaces may have overrepresented educated ‎and digitally literate women, limiting the representativeness and generalizability of findings to ‎the broader UAE female population. As recruitment was limited to two emirates (Sharjah and ‎Dubai) using non-probability convenience sampling, the sample may not be fully representative ‎of all UAE women, and findings should not be generalized beyond similar populations. Third, ‎the cross-sectional design precludes any causal inference; all findings should be interpreted as ‎associations only. Furthermore, although the questionnaire captured key aspects of knowledge ‎and behavioral practice, it did not fully account for cultural, social, or economic factors that ‎may influence osteoporosis prevention. Selection bias is also a concern, as women who chose to ‎participate may differ systematically from those who did not. Additionally, the observed score ‎range of 0-14 out of a possible 17 on the modified OKAT suggests that certain items may have ‎been particularly challenging for this population, which may reflect genuine knowledge gaps ‎rather than instrument limitations, consistent with the study’s overall findings of suboptimal ‎osteoporosis knowledge‎.

## Conclusions

This study found that osteoporosis knowledge was significantly associated with ‎vitamin D and calcium supplementation behaviors, but did not correspond with broader ‎preventive practices such as dietary calcium intake or sun exposure in this sample of UAE ‎women. Multinomial logistic regression indicated that adults aged 51-65 years had ‎approximately five times higher odds of high knowledge compared to younger women aged 19-‎‎35 (OR = 5.084, 95% CI (1.769, 14.605), p = 0.003), and adults aged 36-50 had approximately ‎three times higher odds (OR = 3.257, 95% CI (1.266, 8.380), p = 0.014), while education level ‎and ethnicity were not significantly associated with knowledge category. These findings suggest ‎that targeted interventions, particularly aimed at younger women, may be warranted to ‎address knowledge gaps and that behavioral change strategies beyond awareness alone may be ‎needed to improve osteoporosis prevention practices‎.‎

## Appendices

Appendix A: Osteoporosis Questionnaire (English version)

Section 1: Demographics

Please specify your gender:

1)      Female

2)      Male

Please choose the age group that you fit into:

1)      Below 18

2)      19-35

3)      36-50

4)      51-65

5)      above 65

Do you have any prior knowledge of osteoporosis from your education or field of work?

1)      Yes

2)      No

Have you ever been diagnosed with osteoporosis?

1)      Yes

2)      No

Please check your highest level of education:

1)      Did not complete high school

2)      High school diploma

3)      Bachelors

4)      Masters

5)      PhD

What is your ethnicity?

1)      UAE national

2)      Arab (except UAE)

3)      Far East Asian (like the Philippines and China)

4)      South-East Asians (India, Pakistan, Bangladesh, and Sri Lanka)

5)      Caucasians

6)      Other

Which emirate do you reside in?

1)    Sharjah

2)    Ajman

3)    Dubai

4)    Abu Dhabi

5)    Fujairah

6)    Ras Al Khaimah

7)    Umm AlQuwain

Section 2: Osteoporosis Knowledge (17 Questions): Modified Version of the OKAT Tool

Choose the suitable option for each of the following statements:

True

False

I don’t know

8. Osteoporosis leads to an increased risk of bone fractures

9. Osteoporosis usually causes symptoms (e.g., pain) before fractures occur

10. Osteoporosis is more common in men

11. Cigarette smoking increases the risk of osteoporosis

12. A fall is just as important as low bone strength in causing fractures

13. By age 80, the majority of women have osteoporosis

‎14. Any type of physical activity is beneficial for osteoporosis

15. It is easy to tell whether I am at risk of osteoporosis by my clinical risk‎ factors

16. Family history of osteoporosis increases the risk of osteoporosis

17. Having two glasses of milk a day is enough for your calcium intake

18. Sardines and broccoli are good sources of calcium for people who‎ cannot take dairy products

19. Calcium supplements alone can prevent bone loss

20. A high salt intake is a risk factor for osteoporosis

21. There is a small amount of bone loss in the ten years following ‎the onset of menopause

22. Hormone therapy prevents further bone loss at any age after menopause

‎23. There are no effective treatments for osteoporosis available in the UAE

24. A bone mineral density test should be performed monthly to ‎monitor bone loss

Section 3: Preventive Practices

25. How many glasses of milk do you drink in a day?

1)      0

2)      1-2

3)      More than 2

26. Calcium intake:

ِA. How often do you take calcium supplements?

1)      Never

2)      Once daily

3)      Every other day

4)      Once weekly

5)      Once every month

B. What stops you from taking more calcium?

1)      Lactose intolerance

2)      Unavailability of supplements

3)      Unclarity about the required calcium intake

4)      Not allowed to take with certain medications

5)      Other

27. How often do you eat vitamin D-rich foods (such as liver, mushrooms, spinach, fish oils, eggs)?

1)    Never

2)    Once daily

3)    Every other day

4)    Once weekly

5)    Once every month

28. How often do you take vitamin-D supplements?

1)    Never

2)    Once daily

3)    Every other day

4)    Once weekly

5)    Once every month

29. Exercise:

A. Do you exercise for 20 minutes or more daily?

1)      Yes

2)      No

B. What do you think would motivate you to do more exercise?

1)      Motivational social media posts

2)      Gyms that are nearly located

3)      Awareness campaigns

30. Do you spend up to 15 minutes in the sun daily?

1)      Yes

2)      No

31. Do you think there is a need for more preventive programs regarding ‎osteoporosis in the UAE?‎

1)      Yes

2)      No

32. If you have any previous knowledge about Osteoporosis, please tick below the answer(s) that best describes your source of knowledge.

1)      TV

2)      Social media

3)      Articles\websites

4)      Campaigns

5)      Newspapers

6)      Other

Appendix B: Osteoporosis Questionnaire (Arabic version)

القسم الأول: البيانات الديموغرافية

1. يرجى تحديد جنسك:

1.    أنثى

2.     ذكر

2. ما هي فئتك العمرية؟

تحت ال18

19-35

51-65

فوق ال65

3. هل لديك أي معرفة مسبقة بهشاشة العظام من تعليمك أو مجال عملك؟

1.    نعم

2.    لا

4. هل سبق أن تم تشخيصك بهشاشة العظام؟

1.    نعم

2.    لا

5. ما هو تحصيلك العلمي:

1.    لم أكمل المدرسة الثانوية

2.    شهادة الثانوية العامة

3.    شهادة البكالوريوس

4.    شهادة الماجستير

5.    شهادة الدكتوراه

6. إلى أي عرقٍ تنتمي؟

1.    مواطنة إماراتية

2.    عربية (باستثناء الإمارات العربية المتحدة)

3.    شرق آسيا (مثل الفلبين والصين)

4.    جنوب شرق آسيا (الهند وباكستان وبنغلاديش وسريلانكا)

5.    الدول الأوروبية

6.    أخرى

7. ما هي الإمارة التي تقيمين فيها؟

1.    الشارقة

2.    عجمان

3.    دبي

4.    أبو ظبي

5.    الفجيرة

6.    رأس الخيمة

7.    أم القيوين

القسم الثاني: المعرفة حول هشاشة العظام

ا*لرجاء اختيار الخيار المناسب لكلٍ من العبارات التالية:*

صحيح

خطأ

لا أعلم

8.    تؤدي هشاشة العظام إلى زيادة خطر الإصابة بكسور العظام

9. عادة ما تسبب هشاشة العظام أعراضاً (مثل الألم) قبل حدوث الكسور

10. هشاشة العظام أكثر شيوعاً عند الرجال

11. يزيد تدخين السجائر من خطر الإصابة بهشاشة العظام

12.  تتساوى نسبة الكسور بسبب السقوط مع نسبة الكسور بسبب انخفاض كثافة العظام

13. بحلول سن الثمانين، تعاني غالبية النساء من هشاشة العظام

14. ممارسة الرياضة بجميع أشكالها مفيدة لهشاشة العظام

15. سأعرف مدى نسبة خطورة إصابتي بهشاشة العظام من عوامل الخطر السريرية

16. يزيد التاريخ العائلي لهشاشة العظام من خطر الإصابة به

17. يكفي شرب كأسين من الحليب يوميًا للحصول على الكالسيوم الكافي

18. تعتبر السردين والبروكلي مصادر جيدة للكالسيوم للأشخاص الذين لا يستطيعون تناول منتجات الألبان

19. مكملات الكالسيوم وحدها يمكن أن تمنع فقدان كثافة العظام

20. إن تناول كميات كبيرة من الملح هو عامل خطر لهشاشة العظام

21. هناك قدرٌ ضئيلٌ من فقدان كثافة العظام في السنوات العشر التالية لظهور انقطاع الطمث

22. بعد انقطاع الطمث (في أي عُمرٍ كان)، يمنع العلاج الهرموني المزيد من فقدان كثافة العظام

23. لا توجد علاجات فعالة لهشاشة العظام متوفرة في الإمارات العربية المتحدة

24. يجب إجراء اختبار كثافة المعادن في العظام شهريًا لمراقبة فقدان العظام

القسم الثالث: الممارسات الوقائية

25. كم كأس من الحليب تشربين في اليوم؟

1.    1

2.    1-2

3.    أكثر من 2

26. تناول الكالسيوم

a.    كم مرة تتناولين مكملات الكالسيوم؟

1.    ‎أبداً

2.    ‎مرة واحدة يوميا

3.    ‎‎مرة كل يومين

4.    ‎‎مرة واحدة أسبوعيا

5.    ‎‎مرة كل شهر

b.    ما الذي يمنعكِ من تناول المزيد من الكالسيوم؟

1.    حساسية اللاكتوز

2.    ‎‎عدم توافر المكملات

3.    ‎‎عدم الوضوح بشأن كمية الكالسيوم ‏المطلوبة

4.    ‎‎غير مسموح بتناوله مع بعض الأدوية

5.    ‎‎أسباب أخرى

27.    كم مرة تأكلين الأطعمة الغنية بفيتامين د (مثل الكبد والفطر والسبانخ وزيوت السمك والبيض)؟

1.    أبداً

2.    مرة واحدة يوميا

3.    مرة كل يومين

4.    مرة واحدة أسبوعيا

5.    مرة كل شهر

28.    كم مرة تتناولين مكملات فيتامين د؟

1.    أبداً

2.    مرة واحدة يوميا

3.    مرة كل يومين

4.    مرة واحدة أسبوعيا

5.    مرة كل شهر

29. ممارسة الرياضة

a.    هل تمارسين الرياضة لمدة 20 دقيقة أو أكثر يوميًا؟

1.    ‎نعم

2.    لا

b.    ما الذي يحفزكِ على ممارسة المزيد من التمارين الرياضية؟

1.    منشورات تحفيزية على وسائل التواصل ‏الاجتماعي

2.    ‎‎ صالات رياضية قريبةُ الموقع

3.    حملات توعوية

30.    هل تقضين ما يصل إلى 15 دقيقة في الشمس يوميًا؟

1.    نعم

2.    لا

31.    هل تعتقدين أن هناك حاجة للمزيد من البرامج الوقائية فيما يتعلق بمرض هشاشة العظام في دولة الإمارات العربية المتحدة؟

1.    نعم

2.    لا

32.    إذا كانت لديك أي معرفة سابقة بمرض هشاشة العظام ، فيرجى اختيار الإجابة (أو الإجابات) التي تصف مصدر معرفتك.

1.    التلفاز

2.    وسائل التواصل الاجتماعي

3.    المقالات \ المواقع

4.    الحملات

5.    الصحف

6.    مصادر أخرى
